# Impact of Social Deprivation on Diagnosis, Management and Outcome of Chronic Inflammatory Demyelinating Polyneuropathy at a Tertiary UK Centre

**DOI:** 10.1111/jns.70054

**Published:** 2025-08-13

**Authors:** Zeinab Rajabally, Mahmoud A. Mohamed, Lydia Spencer, Niraj Mistry, Yusuf A. Rajabally

**Affiliations:** ^1^ Department of Neurology University Hospitals Birmingham UK; ^2^ Aston Medical School Aston University Birmingham UK

**Keywords:** chronic inflammatory demyelinating polyneuropathy, diagnosis, disability, index of multiple deprivation, outcome, social, treatment

## Abstract

**Background:**

Whether social deprivation may affect diagnosis, management, and outcomes of subjects with chronic inflammatory demyelinating polyneuropathy (CIDP) is unknown.

**Methods:**

We conducted a retrospective study of subjects with CIDP attending University Hospitals Birmingham, UK. Demographics, clinical characteristics, treatment data, post‐treatment outcomes and Index of Multiple Deprivation 2019 were collected. Postcodes were categorised in local vs. non‐local and travelling distances to the hospital were ascertained.

**Results:**

We included 155 consecutive subjects with CIDP. Mean age was 62.2 years (SD: 15.1). Male to female ratio was 1.67:1. One‐hundred and eighteen subjects (76.1%) had typical CIDP. Greater pre‐treatment disability was independently associated with greater social deprivation (*p* = 0.031) and longer pre‐treatment disease duration (*p* = 0.001). Neither use of high‐cost first‐line therapies, nor immunosuppressant usage, were associated with social deprivation. Post‐treatment outcomes were not associated with social deprivation. Greater social deprivation was independently associated with younger age (*p* = 0.002), having a local post‐code (*p* = 0.001) and living closer to the hospital (*p* < 0.001). Subjects from the two most socially deprived deciles were younger (*p* = 0.025) and more disabled pre‐treatment (*p* = 0.028) than those from the two least deprived deciles. Significantly fewer tertiary referrals were received for the two most socially deprived deciles compared to the two least deprived deciles (9.9% vs. 31.3%; *p* = 0.001).

**Conclusions:**

Despite a publicly funded healthcare system with universal access, social deprivation independently contributed to greater pre‐treatment disability in subjects with CIDP in this UK cohort. Social deprivation did not impact on treatments administered and post‐treatment outcomes but may have influenced tertiary referral decisions to our centre.

## 1 Introduction

Social deprivation measures the social well‐being of a population, which may impact upon the diagnosis, management, and outcome of disease [1]. It is determined by a number of factors, including educational level, employment status, and access to healthcare facilities. To date, there have been few studies of the impact of social deprivation on neuropathic disorders [2, 3]. To our knowledge, none have focused on inflammatory neuropathies and on chronic inflammatory demyelinating polyneuropathy (CIDP), in particular.

In the UK, the National Health Service (NHS) offers publicly funded, universal access healthcare based on clinical need, free of charge at the point of care. Relationships, in some cases complex, of social deprivation have been described in the UK and within the setting of NHS‐delivered care for multiple common conditions such as psychotic disorders [4], attention deficit hyperactivity disorder (ADHD) [5], multiple sclerosis (MS) [6] or prostatic cancer [7]. On the other hand, no impact of social deprivation was reported for bariatric surgery outcomes [8] or pulmonary arterial hypertension [9]. The latter study contradicted previous analyses performed in the United States [10] and China [11], which may suggest a possible beneficial effect of the UK healthcare system on the impact of social inequality in this rare disorder. It is possible that such favourable effects of a free and universal access healthcare system may be more evident for uncommon conditions. Whether this may similarly be the case for CIDP, a rare disease of comparable incidence to that of pulmonary arterial hypertension [12, 13], is unknown.

We proposed to conduct a retrospective study of patients diagnosed with CIDP attending our service to evaluate the effects of social deprivation on diagnosis, disability at presentation, treatment modalities, and post‐treatment outcomes. We also aimed to determine the impact of postcode location as well as travelling distance of patients' place of residence to our hospital on diagnosis, management, and outcome, as well as on out‐of‐area tertiary referral rates to our centre.

## 2 Methods

We retrospectively reviewed electronic case records of all consecutive subjects presenting with a diagnosis of ‘CIDP’ or ‘possible CIDP’, of all subtypes, meeting the requirements of the European Academy of Neurology/Peripheral Nerve Society (EAN/PNS) updated Guidelines of 2021 [14], attending the Inflammatory Neuropathy Service, University Hospitals Birmingham, United Kingdom. We included subjects who had received treatment for CIDP for at least 12 months in order to meaningfully evaluate treatment effects on outcomes. Included subjects had first attended our service between January 2014 and May 2024.

We determined demographics, CIDP disease subtype, disease duration from symptom onset to time of treatment initiation at our centre, treatment with first‐line high‐cost therapies (immunoglobulins and/or plasma exchange) at any stage of treatment, and presence of comorbidities with functional impact. Pre‐treatment disability was evaluated through the Overall Neuropathy Limitation Score (ONLS) [15]. The ONLS scale is systematically used at all clinic attendances in our practice (five points for upper limb score, seven points for lower limb score; optimal score of 0). Post‐treatment outcome was defined as the best level of function achieved after at least 12 months of active treatment and determined through the post‐treatment ONLS, the post‐treatment inflammatory Rasch‐built Overall Disability Scale (I‐RODS) [16], as well as through the ‘CIDP Treatment‐Response Category’ (‘CT‐RC’), which we previously proposed, constructed on an exclusively result‐based categorical classification of response [17]. The CT‐RC is graded 1–5: (i) CT‐RC 1: ‘complete response’ (corresponding to full recovery) (ii) CT‐RC 2: ‘good partial response’ (equivalent to at least having the ability to do all common self‐care tasks and ability to walk without aid), (iii) CT‐RC 3: ‘moderate partial response’ (equivalent to at least having the ability to do most but not all common self‐care tasks and ability to walk with unilateral support) (iv) CT‐RC 4: ‘poor partial response’ (equivalent to at least having purposeful upper limb movements without the ability to perform any common self‐care task and ability to walk with bilateral support) (v) CT‐RC 5: ‘unresponsive’ (corresponding to no or no meaningful change from pre‐treatment level).

The English Index of Multiple Deprivation 2019 (IMD 2019) provides a set of relative measures of deprivation for small geographical areas based on seven domains: (i) income deprivation, (ii) employment deprivation, (iii) education, skills, and training deprivation, (iv) health deprivation and disability, (v) crime, (vi) barriers to housing and services, and (vii) living environment deprivation [18]. Each domain was based on a basket of indicators, and each basket was based on the most recent data available. The IMD 2019 combines information from the seven principal domains. Respective weights for each of the seven above‐mentioned domains were 22.5%, 22.5%, 13.5%, 13.5%, 9.3%, 9.3% and 9.3%, respectively. Each domain was constructed separately from the component indicators, and each area was assigned a domain score representing a combination of these indicators, and each area was then ranked according to this domain score. The domain ranks were then transformed to an exponential distribution before being combined to produce an overall relative measure of deprivation, expressed as a rank and decile for each area, known as the IMD [18]. The lowest IMD decile (Decile 1) corresponds to the 10% most deprived areas and the highest (Decile 10) to the 10% least deprived. For the current analysis, addresses were considered at the time of initial presentation for medical attention for symptoms of CIDP. We used house number, street name and postcode through the Geographic Data Service Map maker [19], cross‐referenced with Google Maps to establish the corresponding IMD 2019 decile for each included subject. Postcodes were categorised into two groups: local (Birmingham) and non‐local (non‐Birmingham), and travelling distances from the place of residence to our hospital were determined in each case.

We ascertained the associations of IMD 2019 decile, postcode category and travelling distance to the hospital with (i) demographics, (ii) CIDP subtype, (iii) pre‐treatment disease duration, (iv) pre‐treatment ONLS, (v) post‐treatment ONLS, (vi) post‐treatment I‐RODS, (vii) CT‐RC, (viii) treatment with high‐cost first‐line therapy for CIDP with immunoglobulins and/or plasma exchange, and (ix) treatment with immunosuppressant agent(s). In order to better characterise and understand potential differences between extreme ends of the spectrum of social deprivation in our cohort, we also compared disease characteristics, treatment modalities and outcomes of subjects from the two most deprived and two least deprived IMD 2019 deciles.

Statistical analyses were performed with SPSS 28.0 (Armonk, USA). Comparison of proportions was performed by Fisher's exact tests; comparison of means was performed by independent Mann–Whitney *U* tests. Correlations were performed by Spearman's rank correlation tests. In view of the exploratory nature of this analysis, corrections for multiple correlations were not performed. Independent associations were sought through linear regression, considering in the models the relevant co‐variates demonstrating significant association through Spearman's rank correlation. Significance was set at *p* < 0.05.

This study was reviewed and approved at our institution as a retrospective clinical audit of the impact of socio‐economic factors on diagnosis, management and outcome in patients with CIDP attending our service (CARMS‐22724, 16 January 2025). Audit does not require Ethics Committee approval in the UK.

## 3 Results

### 3.1 Baseline Characteristics

We included 155 consecutive subjects meeting EAN/PNS criteria for CIDP or possible CIDP, having attended our service between January 2014 and May 2024 and having been followed up for at least 12 months. The main characteristics of included subjects are summarised in Table 1. There were 58 females and 97 males (ratio of 1:1.67). Mean age was 62.2 years (S.D.: 15.1), and mean disease duration pre‐treatment initiation at our centre was 37 months (SD: 55). A total of 100 and 18 subjects (76.1%) had typical CIDP, 24 (15.5%) had variant focal/multifocal CIDP, 4 (2.6%) had motor CIDP and 7 (4.5%) had sensory CIDP. Forty‐three (27.7%) had an associated comorbidity (cardio‐respiratory, rheumatological or neurological) impacting on physical function, in addition to CIDP. Mean pre‐treatment ONLS was 5.56 (SD: 2.7), and best achieved post‐treatment ONLS after at least 12 months of therapeutic intervention was 2.17 (SD: 1.9). Mean raw post‐treatment I‐RODS was 35.6 (SD: 10.4). A total of 45 subjects (29%) were classified in CT‐RC 1, 59 (38.1%) in CT‐RC 2, 14 (9%) in CT‐RC 3, 22 (14.2%) in CT‐RC 4 and 15 (9.7%) in CT‐RC 5. Post‐treatment ONLS, post‐treatment I‐RODS, and CT‐RC were highly intercorrelated.

**TABLE 1:** Demographic, clinical and socio‐economic characteristics and geographical location of residence of 155 subjects with CIDP attending University Hospitals Birmingham, UK.

Mean current age‐years (SD)	59.3 (12.6)
Gender F:M (ratio)	58:97 (1:1.67)
Mean pre‐treatment disease duration‐months (SD)	37 (55)
CIDP disease subtype	– Typical: 118/55 (76.1%) – Variant focal/multifocal: 24/155 (15.5%) – Motor: 4/155 (2.5%) – Sensory: 7/155 (4.5%)
Mean pre‐treatment ONLS (SD)	5.56 (2.7)
Mean post‐treatment ONLS (SD)	2.17 (1.9)
Mean raw post‐treatment I‐RODS (SD)	35.6 (10.4)
CT‐RC distribution	– CT‐RC 1: 45/155 (29%) – CT‐RC 2: 59/155 (38.1%) – CT‐RC 3: 14/155 (9%) – CT‐RC 4: 22/155 (14.2%) – CT‐RC 5: 15/155 (9.7%)
IMD 2019 decile distribution	– Decile 1: 11% Decile 6: 7.1% – Decile 2: 7.7% Decile 7: 15.5% – Decile 3: 8.4% Decile 8: 7.7% – Decile 4: 11.6% Decile 9: 12.3% – Decile 5: 12.9% Decile 10: 5.8%
Postcode location distribution	Local (Birmingham): 64/155 (41.3%) Non‐local (non‐Birmingham): 91/155 (58.7%)
Travelling distance to hospital	≤ 30 miles: 118 subjects (76.1%) > 30 miles: 37 subjects (23.9%)
Mean travelling distance to hospital‐miles (SD)	27.75 (38.38)

**FIGURE 1:**
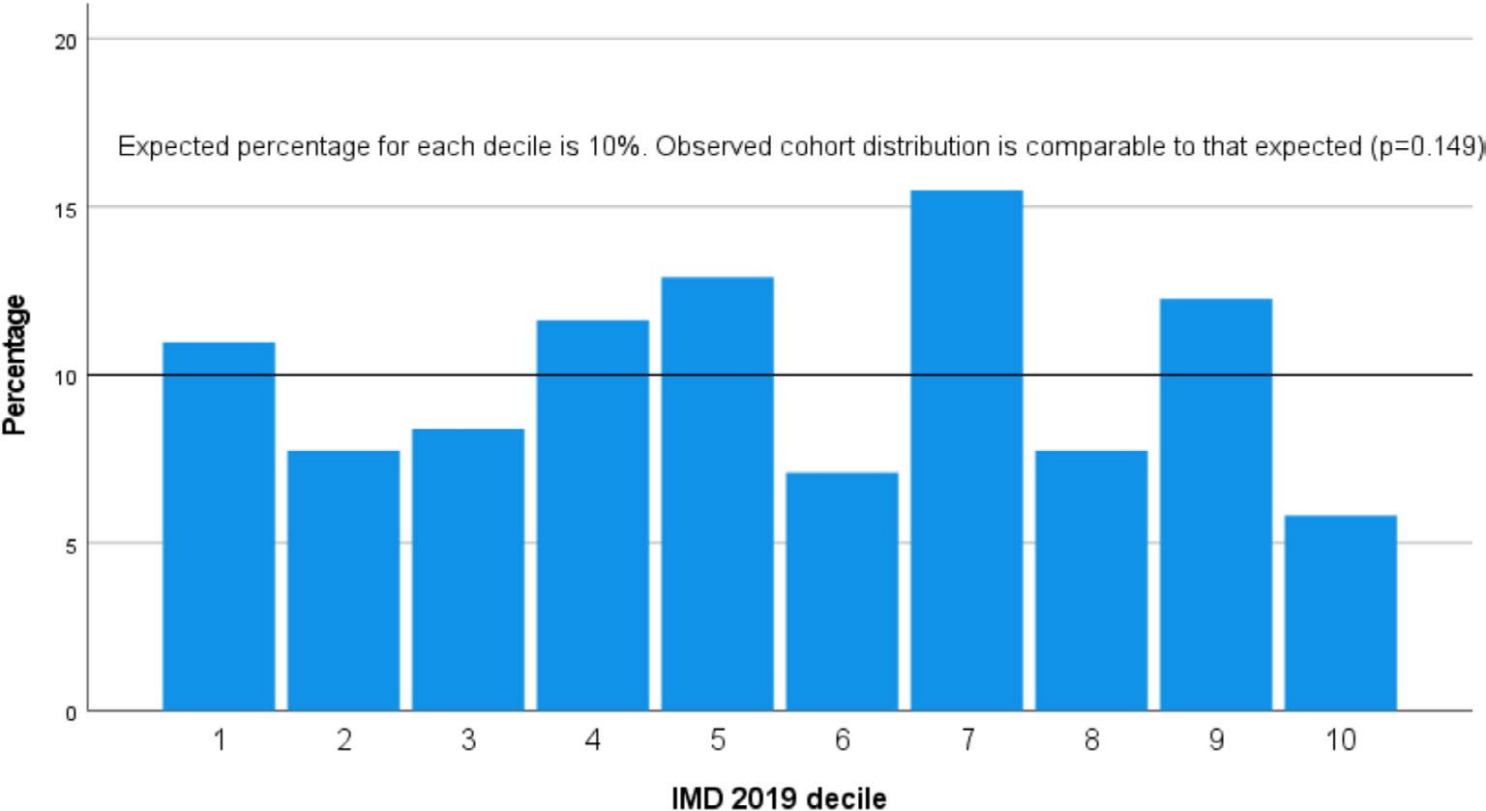
Observed IMD 2019 decile distribution in 155 consecutive subjects with CIDP attending University Hospitals Birmingham, UK.

The full range of IMD 2019 deciles was observed in the cohort (Decile 1: 11%; Decile 2: 7.7%; Decile 3: 8.4%; Decile 4: 11.6%; Decile 5: 12.9%; Decile 6: 7.1%; Decile 7: 15.5%; Decile 8: 7.7%; Decile 9: 12.3%; Decile 10: 5.8%) (Figure 1). This was consistent with the distribution observed in the population of England in 2020, as per the division into 10 equal‐sized groups of the 32 844 ‘Lower‐layer Super Output Areas’ in England, with Decile 1 representing the most deprived 10% of areas nationally and Decile 10 representing the 10% least deprived of areas nationally (*p* = 0.149) [18]. A total of 64 subjects (41.3%) had a local postcode, and 91 (58.7%) had a non‐local postcode. One hundred and eighteen (76.1%) subjects lived ≤ 30 miles from the hospital, and 37 (23.9%) subjects lived > 30 miles from the hospital. Mean travelling distance from the hospital was 27.75 miles or 46.35 km (range: 1.1–274; SD: 38.38).

### 3.2 Correlates of IMD 2019 Decile

IMD 2019 decile correlated with age (*p* = 0.008) and inversely with (i) total pre‐treatment ONLS (*p* = 0.021), (ii) having a local postcode (*p* = 0.001), and (iii) travelling distance to the hospital (*p* < 0.001) (Table 2). There were no correlations of IMD 2019 decile with total post‐treatment ONLS, post‐treatment I‐RODS, CT‐RC, presence of associated comorbidities with functional impact, treatment with high‐cost therapies, or treatment with immunosuppressant(s). Hence, greater social deprivation was associated with greater pre‐treatment disability, younger age, having a local postcode, and living closer to the hospital.

**TABLE 2:** Spearman's rank correlations for IMD 2019, postcode location and travelling distance to the hospital with relevant co‐variables in 155 consecutive subjects with CIDP attending University Hospitals Birmingham, UK.

	Age	Pre‐treatment disease duration	Pre‐treatment ONLS	Post‐treatment ONLS	Post‐treatment I‐RODS	CT‐RC	Comorbidities with functional impact	Use of IVIg	Use of PLEX	Use of IS	IMD 2019	Postcode location	Travelling distance to the hospital
IMD 2019	*ρ* = 0.211 *p* = 0.008	NS	*ρ* = −0.186 *p* = 0.021	NS	NS	NS	NS	NS	NS	NS	—	*ρ* = −0.256 *p* = 0.001	*ρ* = −0.335 *p* < 0.001
Postcode location	NS	*ρ* = −0.242 *p* = 0.002	*ρ* = 0.194 *p* = 0.015	NS	NS	NS	NS	NS	NS	NS	*ρ* = −0.256 *p* = 0.001	—	*ρ* = −0.732 *p* < 0.001
Travelling distance to the hospital	NS	*ρ* = 0.259 *p* = 0.001	*ρ* = −0.165 *p* = 0.04	NS	NS	NS	NS	NS	NS	NS	*ρ* = −0.335 *p* < 0.001	*ρ* = −0.732 *p* < 0.001	—

Abbreviations: IMD 2019, Index of Multiple Deprivation 2019 decile; I‐RODS, inflammatory Rasch‐built Overall Disability Scale; IS, immunosuppression; IVIg, intravenous immunoglobulins; NS, non‐significant; ONLS, overall neuropathy limitation score; PLEX, plasma exchange.

### 3.3 Correlates of Postcode

**FIGURE 2:**
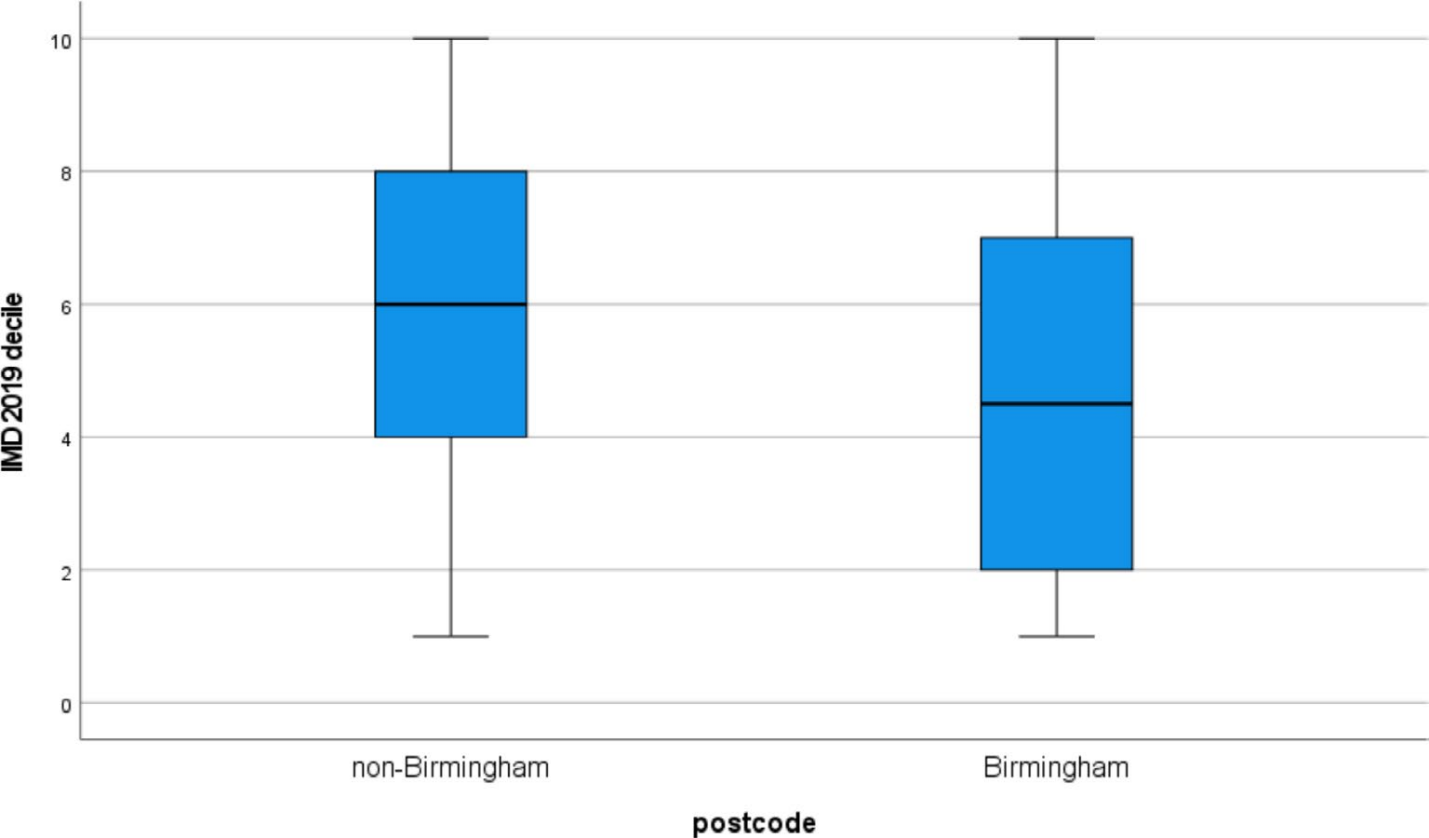
Boxplot of IMD 2019 distribution in non‐Birmingham and Birmingham postcodes for 155 subjects with CIDP attending University Hospitals Birmingham, UK.

Having a local postcode correlated with total pre‐treatment ONLS (*p* = 0.015), and inversely with IMD 2019 decile (*p* = 0.001) and pre‐treatment disease duration (*p* = 0.002) (Table 2), and with travelling distance to the hospital (*p* < 0.001). IMD 2019 decile distribution across postcode is shown in Figure 2. There were no correlations of having a local postcode with age, total post‐treatment ONLS, post‐treatment I‐RODS, CT‐RC, presence of associated comorbidities with functional impact, or treatment with the first‐line high‐cost therapies. Hence, a local postcode was associated with greater social deprivation, greater pre‐treatment disability and shorter pre‐treatment disease duration.

### 3.4 Correlates of Travelling Distance to the Hospital.

**FIGURE 3:**
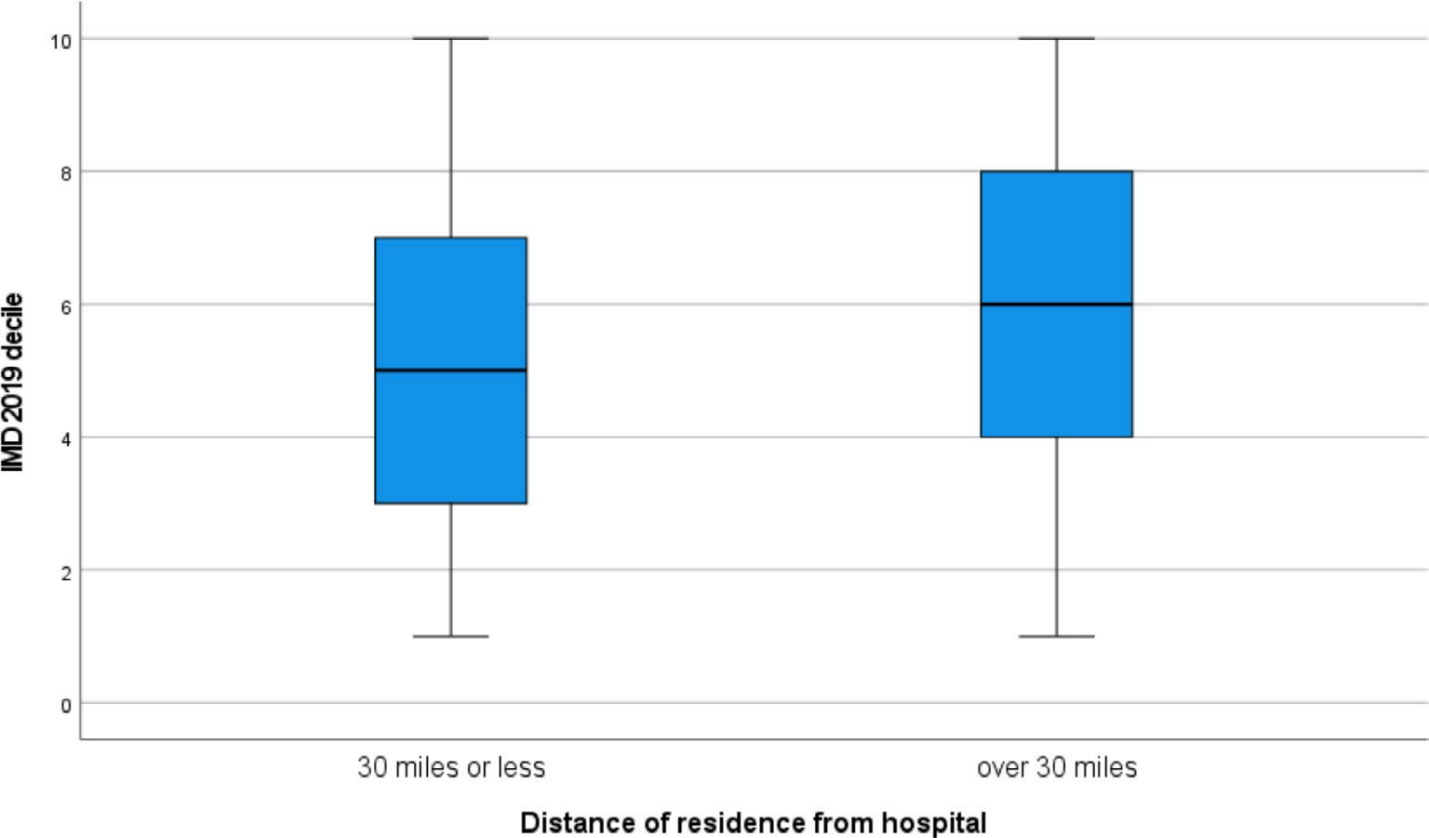
Boxplot of IMD 2019 distribution in subjects living ≤ 30 miles and ≥ 30 miles from the hospital from 155 subjects with CIDP attending University Hospitals Birmingham, UK.

Travelling distance to the hospital correlated with IMD 2019 decile (*p* < 0.001) and pre‐treatment disease duration (*p* = 0.001). An inverse correlation was found with total pre‐treatment ONLS (*p* = 0.04) (Table 2) and having a local postcode (*p* < 0.001). IMD 2019 decile distribution according to travelling distance (≤ 30 miles or > 30 miles) is shown in Figure 3. Travelling distance was not associated with age, total post‐treatment ONLS, post‐treatment I‐RODS, CT‐RC, presence of associated comorbidities with functional impact, or treatment with first‐line high‐cost therapies for CIDP. Hence, living further away from the hospital was associated with less social deprivation, longer treatment delay, and less pre‐treatment disability.

### 3.5 Factors Independently Associated With Pre‐ and Post‐Treatment Disability

Linear regression analyses were performed, including in the models the relevant and significant correlates of pre‐ and post‐treatment disability (Table 3). Pre‐treatment ONLS was independently associated with IMD 2019 decile (*p* = 0.03) and pre‐treatment disease duration (*p* = 0.001). No independent association was found with postcode location or travelling distance to the hospital. Post‐treatment ONLS was only independently associated with age (*p* = 0.018). Post‐treatment I‐RODS was independently associated with age (*p* = 0.004) but not with comorbidities with functional impact. CT‐RC was independently associated with age (*p* = 0.009) but not with pre‐treatment disease duration. Hence, greater pre‐treatment disability was independently associated with greater social deprivation and longer pre‐treatment disease duration. On the other hand, worse post‐treatment outcome was consistently only independently associated with older age.

**TABLE 3:** Significance level results of linear regression analyses for independent predictors of pre‐treatment ONLS, post‐treatment ONLS, post‐treatment I‐RODS and CT‐RC amongst relevant co‐variables in 155 consecutive subjects with CIDP attending University Hospitals Birmingham, UK.

	Age	IMD 2019 decile	Pre‐treatment disease duration	Comorbidities with functional impact	Postcode location	Travelling distance to the hospital	Implication
Pre‐treatment ONLS	NA	*p* = 0.03	*p* = 0.001	NA	NS	NS	Worse pre‐treatment disability is independently associated with greater social deprivation and longer pre‐treatment disease duration
Post‐treatment ONLS	*p* = 0.018	NA	NA	NA	NA	NA	Worse post‐treatment disability is only independently associated with older age
Post‐treatment I‐RODS	*p* = 0.004	NA	NA	NS	NA	NA	Worse post‐treatment disability is only independently associated with older age
CT‐RC	*p* = 0.009	NA	NS	NA	NA	NA	Worse post‐treatment disability is only independently associated with older age

Abbreviations: NA, non‐applicable; NS, non‐significant.

### 3.6 Grouped Comparisons Based on Social Deprivation

Findings are summarised in Table 4. In comparison to subjects from the two least deprived IMD 2019 deciles (28 subjects), subjects from the two most deprived deciles (29 subjects) (i) were significantly younger (Mann–Whitney *U* Test; *p* = 0.025), (ii) were significantly more disabled pre‐treatment (Mann–Whitney *U* Test; *p* = 0.028), (iii) were significantly more likely to have a local post code (Fisher's exact test; *p* = 0.001), and (iv) lived significantly closer to the hospital (Mann–Whitney *U* Test; *p* < 0.001). There were no differences between the two groups for pre‐treatment disease duration (Mann–Whitney *U* Test; *p* = 0.132), post‐treatment total ONLS (Mann–Whitney *U* Test; *p* = 0.353), post‐treatment I‐RODS (Mann–Whitney *U* Test; *p* = 0.148), CT‐RC (Mann–Whitney *U* Test; *p* = 0.437), comorbidities with functional impact (Fisher's exact test; *p* = 0.468), treatment with immunoglobulins (Fisher's exact test; *p* = 0.292), treatment with plasma exchange (Fisher's exact test; *p* = 1), or use of immunosuppressants (Fisher's exact test; *p* = 0.67). Comparing referral rates within the group of subjects with non‐local postcodes who had been referred to our service by their local neurologists, significantly fewer tertiary referrals were received for the two most socially deprived deciles compared to the two least socially deprived deciles (9.9% vs. 31.3%; Fisher's exact test; *p* = 0.001). Hence, although younger and more disabled than those in the two least deprived deciles, subjects living out‐of‐area in the two most deprived deciles were significantly less likely to be referred to the tertiary centre.

**TABLE 4:** Grouped comparisons of relevant co‐variables of the two most deprived IMD 2019 deciles versus the two least deprived IMD 2019 deciles in 57 consecutive subjects with CIDP attending University Hospitals Birmingham, UK.

Co‐variate studied	Age	Pre‐treatment disease duration	Pre‐treatment ONLS	Post‐treatment ONLS	Post‐treatment I‐RODS	CT‐RC	Comorbidities with functional impact	Use of IVIg	Use of PLEX	Use of IS	Postcode location	Travelling distance to the hospital
Co‐variate data type	Continuous	Continuous	Continuous	Continuous	Continuous	Continuous	Categorical	Categorical	Categorical	Categorical	Categorical	Continuous
Test performed	Mann–Whitney *U* Test	Mann–Whitney *U* Test	Mann–Whitney *U* Test	Mann–Whitney *U* Test	Mann–Whitney *U* Test	Mann–Whitney *U* Test	Fisher's exact test	Fisher's exact test	Fisher's exact test	Fisher's exact test	Fisher's exact test	Mann–Whitney *U* Test
Significance	*p* = 0.025	NS	*p* = 0.028	NS	NS	NS	NS	NS	NS	NS	*p* = 0.001	*p* < 0.001
Implication	Most socially deprived subjects are younger	Similar in both groups	Most socially deprived subjects are more disabled pre‐treatment	Similar in both groups	Similar in both groups	Similar in both groups	Similar in both groups	Similar in both groups	Similar in both groups	Similar in both groups	Most socially deprived subjects live locally	Most socially deprived subjects live closer to the hospital

## 4 Discussion

Although the effects of social deprivation are well‐documented for neurological conditions [20], they have, to our knowledge, not been studied in UK practice for CIDP. In the current study, we found that greater pre‐treatment disability was independently associated with greater social deprivation in subjects with CIDP attending our centre. We concurrently found that subjects living locally and in proximity to our hospital were younger and more socially deprived, in keeping with previous studies of inner‐city populations [21]. Potential explanations for greater pre‐treatment disability in more socially deprived individuals are multiple and may include structural causes such as variable primary care access [22], inadequate local referral pathways for neurology outpatient services [23], and longer waiting times in emergency departments of larger centres treating more complex cases and serving deprived populations, such as ours [24]. In keeping with our findings, longer hospital waiting times have previously been found to impact more adversely on deprived populations [25, 26, 27]. Physician‐related factors such as therapeutic inertia, defined as the absence of treatment initiation or intensification when treatment goals are unmet [28], may have played a role in the initial stages of medical evaluation for CIDP, as shown in previous UK studies of subjects with diabetes [29, 30]. Patient‐related factors may also have had an impact through delay in seeking medical attention despite progressive disability, due to a lower educational level and reduced health awareness [31]. In addition, although we found that comorbidities with functional impact were as common in the two least deprived compared to the two most deprived deciles, it is noteworthy that the latter group was significantly younger and therefore had an unexpectedly high rate of comorbidity for age, which may have contributed to their greater pre‐treatment disability. This is in keeping with projections of excess comorbidity in the most deprived in England in the coming decades [32]. Furthermore, the presence of other comorbidities not assessed in this study, such as mental health conditions, more common in socially deprived populations [25, 33], may have represented another contributory factor. Social deprivation may otherwise have also impacted upon pre‐treatment status in this cohort through multiple other factors such as low diet quality [34], strenuous work [35], or poor access to green spaces and to opportunities to exercise [36], which may adversely affect weight, cardiovascular and joint health, and, as a result, physical activity and well‐being.

We found no association between social deprivation or geographical place of residence with the use of high‐cost first‐line treatments or the use of immunosuppressive therapy. This suggests equity of access to CIDP first‐line treatments, as well as to immunosuppression when indicated. In relation to immunoglobulin treatment, this may be partly explained by the free and universal access to our service through primary or secondary care referral, as per NHS policy, without restriction based on place of residence. National commissioning guidelines otherwise regulate the prescription of immunoglobulins based exclusively on clinical indication [37]. With regard to plasma exchange, the setting up within our unit in 2020 of an outpatient service for neurology patients enabled performing centrifugal plasma exchange through peripheral venous access instead of central venous lines. This service, in practice, allowed wider and more uniform, as well as likely more equitable, access to subjects with CIDP requiring plasma exchange, as suggested in other UK centres by recent studies [38].

No association was found between social deprivation and post‐treatment outcome, which was studied through three different measures. Despite greater baseline disability pre‐treatment, the effects of immunomodulatory therapy in more socially deprived individuals, therefore, appear to have allowed clinical amelioration to match outcomes of those less socially deprived and less disabled at baseline. This may be partly explained by the absence of an association of social deprivation with pre‐treatment disease duration, which we and others have previously found to impact on outcome in newly diagnosed CIDP [39, 40].

The associations of lesser social deprivation with non‐local postcodes and longer travelling distances to our hospital imply a greater likelihood of access to tertiary referral to our regional centre for less socially deprived subjects. The significantly higher tertiary referral rate in this cohort for patients in the two least deprived deciles compared to the two most deprived deciles also supports this conclusion. Similar findings have been reported previously in several other medical subspecialities [41, 42, 43], and both physician‐related biases including anchoring bias [44] and therapeutic inertia [45], as well as patient‐related factors such as low health literacy, which is linked to social deprivation and found to result in poorer use of health services, may have been implicated [46].

Our study has several limitations, including its retrospective, single‐centre design and a relatively small number of included subjects, explained mainly by the low prevalence of CIDP. Eventual time‐related differences in associations between social deprivation and disease characteristics, treatment and outcomes could, as a result, not be ascertained. Lack of consideration of psychiatric comorbidities also represents an important drawback. In addition, our findings relate to healthcare provision within the UK and may not be applicable to other countries with different systems. However, we believe the results are of relevance to many nations with similar, predominantly publicly funded healthcare. Otherwise, although multiple outcome measures were considered as available from electronic patient records for post‐treatment evaluations, we were unable to determine the effects of social deprivation on health‐related quality of life (HR‐QoL) measures and patients' global impression of change.

In conclusion, the results of this analysis indicate that social deprivation impacted adversely on pre‐treatment disability and access to tertiary referral in this UK CIDP cohort, despite a healthcare system of universal access and free of charge. Reassuringly, no impact of social deprivation could be ascertained on the use of high‐cost therapies or on post‐treatment outcomes. We believe it is likely the UK healthcare infrastructure contributed to the equity observed in our study, as opposed to what has been reported in other similarly rare neurological diseases in countries with different healthcare systems [47, 48]. However, in view of the identified important inequities affecting deprived individuals in our cohort, further study of the effects of social deprivation is needed in rare neuromuscular diseases like CIDP, particularly to improve timely diagnosis and access to specialised centres. Future research will also be of interest in specifically evaluating the impact of social deprivation on improvement of HR‐QoL measures with treatment, as well as more precisely identifying the individual determinants of deprivation which may exert the greatest impact on diagnosis, management and outcomes, with a view to targeted intervention.

## Disclosure

Yusuf A. Rajabally has received consultancy honoraria from Sanofi, Argenx, Janssen, LFB, Polyneuron, Grifols, Takeda, Dianthus, and Vitaccess; has received educational sponsorships from LFB and CSL Behring; and has obtained research grants from LFB. Zeinab Rajabally, Mahmoud A. Mohamed, Lydia Spencer and Niraj Mistry have no disclosures.

## Data Availability

The data that support the findings of this study are available on request from the corresponding author. The data are not publicly available due to privacy or ethical restrictions.
